# Solving the Puzzle: What Is the Role of Progestogens in Neovascularization?

**DOI:** 10.3390/biom11111686

**Published:** 2021-11-12

**Authors:** Zhi Xia, Jian Xiao, Qiong Chen

**Affiliations:** Department of Geriatrics, Xiangya Hospital of Central South University, Changsha 410008, China; xiazhi89@csu.edu.cn (Z.X.); xj2017xh@csu.edu.cn (J.X.)

**Keywords:** progestogens, progesterone, progestins, estrogen, neovascularization, endometrium, cancers

## Abstract

Ovarian sex steroids can modulate new vessel formation and development, and the clarification of the underlying mechanism will provide insight into neovascularization-related physiological changes and pathological conditions. Unlike estrogen, which mainly promotes neovascularization through activating classic post-receptor signaling pathways, progesterone (P4) regulates a variety of downstream factors with angiogenic or antiangiogenic effects, exerting various influences on neovascularization. Furthermore, diverse progestins, the synthetic progesterone receptor (PR) agonists structurally related to P4, have been used in numerous studies, which could contribute to unequal actions. As a result, there have been many conflicting observations in the past, making it difficult for researchers to define the exact role of progestogens (PR agonists including naturally occurring P4 and synthetic progestins). This review summarizes available evidence for progestogen-mediated neovascularization under physiological and pathological circumstances, and attempts to elaborate their functional characteristics and regulatory patterns from a comprehensive perspective.

## 1. Introduction

**Figure 1:**
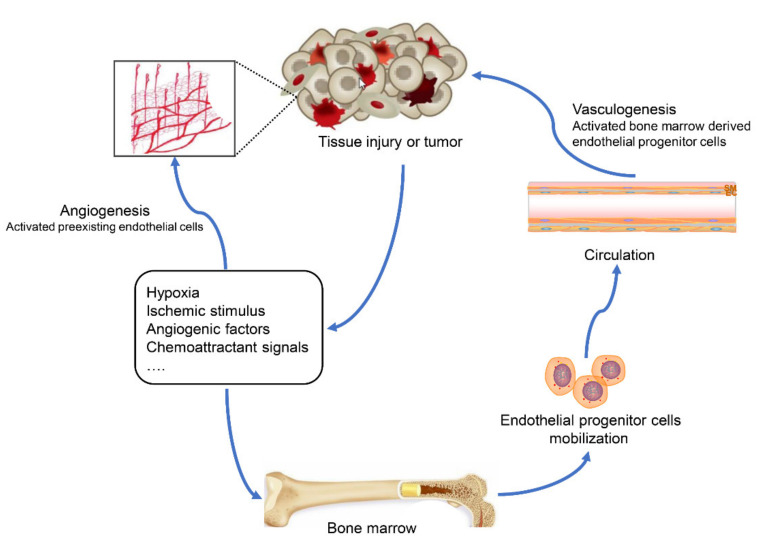
Graphic summarization of neovascularization evoked by tissue injury or tumor. Under the stimulation of hypoxia, ischemic stimulus, angiogenic factors and chemoattractant signals, the two main neovascularization processes are activated. Endothelial cells participate in angiogenesis and form new capillaries from local vessels; endothelial progenitor cells home to target tissues and contribute to primitive vessel formation.

Neovascularization is composed of two conceptions: angiogenesis and vasculogenesis. New capillaries that originate from existing capillaries are called angiogenesis, while vasculogenesis indicates new vessels initially derived from endothelial progenitor cells (EPCs). Although angiogenesis has been proven to be a major part of neovascularization in most situations, these two conceptions coexist and complement each other [1]. Under normal conditions, the vasculature generally remains quiescent, and neovascularization generally occurs in specific physiological and pathological processes, such as the menstrual cycle and tumorigenesis [1,2]. When tissue injury or tumor-associated angiogenic dysregulation happens, angiogenesis can be effectively activated by hypoxia, ischemic stimulus, angiogenic factors and chemoattractant signals, and activated endothelial cells (ECs) from vascular areas acquire proliferative and migrative activities and form angiogenesis sprouting, resulting in vascular morphogenesis from disruptive sites of the basement membrane [3]. Meanwhile, EPCs leave the bone marrow and home to tissue-remodeling sites through the peripheral circulation, and directly incorporate into new vessels, differentiate into mature ECs and produce molecular signals to influence preexisting ECs (Figure 1). Although only a minor player in most instances, vasculogenesis can provide complementary powers especially when there are insufficient conditions for local angiogenesis, contributing to primitive vessel formation [4].

Ovarian sex steroids, mainly estradiol (E2) and progesterone (P4), regulate neovascularization in diverse settings, especially in the endometrium and tumors derived from sex hormone target organs [5,6]. Cholesterol is the common precursor for steroid hormones, and it can be synthesized into P4 and E2 under the actions of enzymes from steroid-generating organs. Based on their lipophilic nature, ovarian sex steroids generally bind to receptors located within the cytoplasm and influence the homing, mobilization and paracrine or juxtacrine signalings of EPCs and ECs. Investigating the role of sex steroids will provide insight into neovascularization-related physiological changes and pathological conditions.

**Figure 2:**
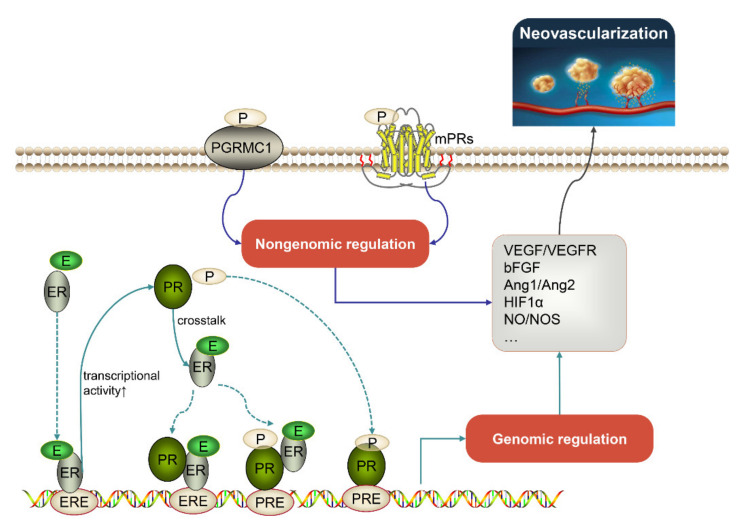
Genomic and nongenomic signaling pathways of progesterone that contribute to regulation of neovascularization. Ang1: angiopoietin1; Ang2: angiopoietin2; bFGF: basic fibroblast growth factor; E: estradiol; ER: estrogen receptor; ERE: estrogen response element; HIF1α: hypoxia inducible factor 1α; P: progesterone; PGRMC1: progesterone membrane component 1; PR: progesterone receptor; PRE: progesterone response element; NO: nitric oxide; mPRs: membrane progestin receptors; NOS: nitric oxide synthase; PD-ECGF: platelet-derived endothelial cell growth factor; VEGF: vascular endothelial growth factor; VEGFR: vascular endothelial growth factor receptor.

P4 mainly functions by crossing the cell membrane and binding to the nuclear progesterone receptor (PR). The P4-PR complex transfers to the nucleus and binds to the special sites of DNA, regulating gene expression. There is an interaction between P4, E2 and their receptors (Figure 2). On the one hand, the estrogen receptor (ER) promotes PR expression at the transcriptional level [7]. On the other hand, PR-A and PR-B, two major PR isoforms involved in P4-mediated actions, exert distinct effects on reprograming E2 signaling; PR-A represses almost 70% of ER-induced transcriptional genes through inhibiting ER genomic recruitment, while PR-B primarily redistributes ER chromatin binding [8,9]. The crosstalk among ER and PR is well-validated. PR, especially PR-B, combines with ER through ER interaction domains to form the PR/ER complex in the presence or absence of P4, which results in a PR/ER crosstalk that can alter gene expression and downstream signaling. Two main crosstalk modes have been reported; the PR/ER complex binds to progesterone response elements (PREs) or other estrogen response elements (EREs), and thus conveys an alteration or enhancement to the original transcriptomes and signaling [10,11]. The tight interaction between PR and ER makes P4 act as a major modifier in E2 effects, and P4 treatment could attenuate ER expression and control ER-mediated transcriptional regulation in line with P4 actions, exhibiting an antagonism on E2 [12,13,14].

In addition to naturally occurring P4, a variety of synthetic progestins are widely used as PR agonists in in vitro or in vivo PR signaling studies, because synthetic progestins possess P4-like structures and effects, as well as great receptor binding affinities and metabolisms [15]. These PR agonists, including naturally occurring P4 and progestins, are classified as progestogens [15]. E2 generally promote neovascularization via binding to ER [16]; however, conflicting observations have been reported in studies on progestogen-mediated neovascularization. Further, E2 combinations can convey a multifaced interaction in neovascularization; progestogens interfere with the expression of E2-induced angiogenic factors and counteract E2 actions in some cases, while the functional synergy between E2 and progestogens has also been confirmed in other studies. Outcomes from diverse experimental models and conditions are yet to clarify the exact role of progestogens.

There is no doubt that progestogens play an indispensable part in neovascularization. The aim of this review is to discuss their influence on neovascularization, and to summarize available evidence on the role of progestogens in physiological and pathological neovascularization, thus yielding an accurate understanding on their functional characteristics and regulatory patterns.

## 2. Downstream Factors Involved in Progestogen-Mediated Neovascularization

The activation of EPCs is of great significance in neovascularization, with EPCs serving as one of the primary sources of ECs constructing the endothelial lining of blood vessels [17,18,19]. Both EPCs and ECs possess the ability to proliferate, adhere and migrate, under the stimulation of pertinent angiogenic factors. Progestogens regulate the expression of cytokines and growth factors in a receptor-dependent manner; through genomic regulation activated by the transcriptional activity of nuclear receptors, or through nongenomic regulation via the second messengers mediated by membrane receptors [20,21]. Therefore, clarifying these downstream factors is a prerequisite for an accurate understanding of progestogen-mediated neovascularization.

### 2.1. Vascular Endothelial Growth Factor (VEGF)

VEGF is a crucial regulator for physiological and pathological neovascularization [22]. Shweiki et al. found that VEGF mRNA tends to be expressed in steroidogenic or steroid-responsive cell types from the female reproductive system, and they first proposed hormones that can regulate VEGF expression to influence the natural angiogenic process [23]. These assumptions were supported by later studies. Progestogens that are combined with E2 induce VEGF expression, leading to EC proliferation in the human endometrial tissue [24,25]. P4 has been confirmed to stimulate VEGF expression, leading to endometrial EC proliferation and capillary-like tube formation [26,27,28,29,30,31]. These observations support the vital role of VEGF in progestogen-mediated neovascularization. In addition to in the endometrium, progestogens promote VEGF expression in skin flaps [32], ovarian tissue [33,34], bovine granulosa cells [35,36], embryonic lung cells [37], granulomas [38] and tumor tissues [39,40,41,42]. However, Keck et al. reported an opposite result, founding that P4 has no impact on the proliferation rate of human umbilical vein endothelial cells (HUVECs), a macrovascular cell model used for endothelial studies in vitro [43]. An anti-angiogenic role of progestogens has been reported in several studies. Okada et al. found that high concentrations of progestins decrease E2-induced VEGF in human endometrial stromal cells [44]. Similar conclusions have been drawn in rat models. Through reducing VEGF expression, P4 effectively ameliorates abnormal angiogenesis in diabetic renal disease and prevents tissue plasminogen activator-induced hemorrhagic transformation in stroke brain tissues [45,46,47]. Although VEGF has been proven to be involved in the downstream of progestogens signaling, conflicting observations suggest that diverse regulatory mechanisms are involved.

PR comprises the main bridge in progestogen-mediated neovascularization. Kim et al. showed that the P4–PR axis is implicated in VEGF physiological changes in the decidual stromal cells, and that PR expression coincides with VEGF in decidual angiogenesis, reporting a rise in expression during pregnancy and a fall during the postpartum period [48]. Hyder et al. confirmed that P4 upregulates VEGF mRNA in a PR-dependent manner [49]. The P4–PR axis is vital to the activation of EPCs. Yu et al. demonstrated that P4-mediated PR improves VEGF expression in EPCs, and thus enhances cell mobilization and tube formation, leading to increases in vessel density and the recovery of neurological function in traumatic brain injury [20,50]. In a previous study, the authors found that P4 regulates EPCs viability through activating PI3K/Akt signaling [51], a validated upstream pathway of VEGF, implying a nongenomic regulation activated by P4. A study found that PR-binding sites were in the promoter regions of the VEGF gene, and that P4-mediated PR regulates VEGF mRNA, confirming that VEGF is directly regulated by the P4–PR axis at the transcriptional level [52]. Meanwhile, some evidence suggests that PR agonist dienogest attenuates VEGF expression in human endometriotic epithelial cell lines [53]. This negative regulation has been confirmed in HUVECs: P4 activates cSrc, a non-receptor tyrosine kinase, to promote the formation of the cSrc-PR complex, which represses downstream RhoA/ROCK signaling and inhibits the proliferation and migration of HUVECs; this effect is abrogated by blocking PR [54]. Obviously, PR is involved in P4-induced VEGF, but its ultimate effect remains unclear. In addition, a study found that P4 promotes the formation of tube-like structures and sprouts of endometrial ECs independent of classic genomic actions by PR [55]. Angio-miRNA might be an important regulator, and Salmasi et al. revealed that P4 treatment promotes the expression of miR-16-5p and miR-17-5p, which bind to the untranslated regions or coding sequence regions of VEGF, and eventually enhances VEGF expression and endometrial EC proliferation [29,30]. Further, membrane progesterone receptors are also implicated in VEGF expression. P4-mediated progesterone membrane component 1 (PGRMC1) induces VEGF expression via PKC signaling, which might promote neovascularization within the retina [56]. Neubauer et al. made a similar conclusion, and they found that P4-mediated PGRMC1 enhances VEGF expression and proliferation of breast cancer cells [57]. However, a study found that the knockout of PGRMC1 and PGRMC2 conveys a repression on luteal vascularization in a mice model, although increasing VEGF mRNA levels were observed in the ovaries and corpora lutea, with the authors proposing that this effect mainly originated from reduced levels of lipocalin 2, resulting in the decreasing expression of matrix metallopeptidase 9 and the activity of VEGF [58].

In addition, P4 influences VEGF angiogenic activity by affecting the levels of vascular endothelial growth factor receptors (VEGFR): VEGFR-1 and VEGFR-2. With rising levels of ovarian sex steroids during pregnancy, VEGF and its receptors are overexpressed in stromal histiocytes and ECs in pregnant tumors of the gingiva [59]. A similar change has been observed in the menstrual cycle; compared to the proliferative phase, a fold increase in VEGFR-1 and vascular density appears in the endometrium during the secretory phase, a stage characterized by high serum P4 concentrations [60], implying a positive correlation between P4 and VEGF receptors. Mandana et al. found that P4 enhances VEGFR levels to ameliorate antiangiogenesis after ovarian stimulation [27]. Subsequent studies confirmed that P4-mediated PR leads to stable increases in VEGF and VEGFR-2, leading to a heightened density of endometrial blood vessels [61], and that P4 antagonist represses VEGFR-2 expression in pregnant uterus tissue [62]. A similar conclusion has been made in studies on breast cancer; the expression of VEGF and VEGFR-2 rises under P4 stimulation, leading to the proliferation of tumor epithelial cells [63]. However, several studies have provided opposite conclusions, finding that P4 withdrawal leads to increases in VEGF, VEGFR-1 and VEGFR-2 in the superficial zone of the macaque or human endometrium, revealing an inhibitory effect on VEGF and its receptors [64,65].

### 2.2. Basic Fibroblast Growth Factor (bFGF)

As a potent angiogenic factor, bFGF binding to its receptor modulates angiogenesis in autocrine and paracrine manners [66]. Chromatin immunoprecipitation (ChIP) and ChIP-sequencing analysis has indicated several PR binding sites in the promoter region of bFGF, and that P4-mediated PR-B upregulates bFGF mRNA, confirming bFGF as the downstream gene in PR signaling [52]. Evidence supports P4′s positive regulation of bFGF. Yuan et al. found increasing blood vessel density and bFGF in granulomas during pregnancy, and that P4 significantly improves the concentration of bFGF in the human monocyte-like U937 cell under the stimulation of lipopolysaccharide [38]. However, medroxyprogesterone acetate (MPA), a synthetic progestin, suppresses bFGF expression and secretion in well-differentiated uterine endometrial tumor cells, while it has no effect on poorly differentiated uterine endometrial tumor cells [67]. In agreement with this finding, P4 and synthetic progestins can inhibit the transcription of bFGF in endometrial tissues, leading to a decreasing vessel density [68].At the same time, P4 exerts an antagonistic effect on E2-induced bFGF [40,69], triggering decreases in bFGF in Ishikawa cells.

### 2.3. Platelet-Derived Endothelial Cell Growth Factor (PD-ECGF)

The increasing expression of PD-ECGF, a strong angiogenic enzyme, parallels the raised concentration of P4 during the late secretory phase in the endometrium, while remains at a normal and stable level during the proliferative phase [70]. A study revealed the physiological concentration of P4 only induces a moderate expression of PD-ECGF in stomal cells, while when combined with transforming growth factor-β1 (TGF-β1), it can induce significant fold changes in epithelial cells, even though P4 or TGF-β1 alone has no influence on PD-ECGF levels [71]. A similar outcome has been observed in uterine endometrial cancer; P4 and its metabolite can lead to a significant increase in PD-ECGF [72]. However, Zavarhei et al. made a controversial conclusion, founding that P4 could suppress PD-ECGF via PR in colorectal cancers [73].

### 2.4. Angiopoietin (Ang)

Ang-1 and Ang-2 are well-studied growth factors involved in angiogenesis; Ang-1 ensures the stability and integrity of the vessel, and Ang-2 influences the matrix contact, EC activity and the permeability of the vessel. Progestogens regulate the two Ang isoforms to maintain the steady function and normal structure of the female reproductive system. Park et al. demonstrated that P4-mediated PR enhances the expression of Ang-2 in human uterine endothelial cells, leading to corresponding vascular remodeling during pregnancy [74]. A paper reported that P4 priming enhances the production of Ang-1 and Ang-2 in large preovulatory follicles, which is important for luteal angiogenesis and normal function [34]. Krikun et al. revealed the mechanism behind abnormal uterine bleeding in long-term progestin-only contraceptives users. MPA promotes Ang-1 in endometrial stromal cells, while it stimulates Ang-2 in endometrial ECs, and progestin-induced local hypoxia diminishes MPA-induced Ang-1, and thus impairs the balance of Ang-1 and Ang-2, resulting in abnormal blood vessels structures [75].

### 2.5. Hypoxia Inducible Factor 1α (HIF1α)

Hypoxia is a contributing factor in progestogen-mediated neovascularization. Long-term progestin-only contraceptives result in endometrial local hypoxia and reperfusion, which damages the progestin-mediated dynamic balance in neovascularization, leading to aberrant angiogenesis, such as abnormally enlarged and fragile microvessels [76]. The interaction between P4 and HIF1α, a hypoxia-induced transcription factor with a proangiogenic function, has been reported in previous studies. HIF1α fluctuates in a menstrual stage-specific manner during corpus luteum development, and HIF1α-mediated VEGF interacts with luteal function, influencing the concentration of P4 and angiogenic status [77]. A microarray analysis for differentiating endometrial stromal cells revealed that P4-mediated PR activates pathways related to blood vessel development and responses to hypoxia, and that VEGF and HIFI1α are important regulators in this process [52], implying a correlation among hypoxia, neovascularization and the P4–PR axis. These results have been confirmed in studies of the bovine granulosa cells and the ewe uterine, which have found that P4 can induce the expression of HIF-1α mRNA and downstream factor VEGF [35,78]. Although publications support the interaction between progestogens and HIF-1α, whether there is a feedback regulation is still unclear.

### 2.6. Nitric Oxide Synthase (NOS)

Previous studies have reported that ECs originating from females are prone to higher endothelial NOS expression, a vital factor in regulating capillary outgrowth and vascular development [79,80], implying a correlation between female sex hormones and endothelial NOS. P4 has been confirmed to enhance endothelial NOS expression through promoting the expression of specificity protein-1, which could form a complex with PR-A and then bind to the promoter region of endothelial NOS [81,82]. Pablo et al. found that P4 promotes nitric oxide (NO) synthesis, EC viability and tube formation, whereas MPA exerts an opposite effect. Irreversible NOS inhibitor significantly reduces P4-induced NO production, and blocking PR partially represses both P4- and MPA-mediated actions; the same inhibitory effect was observed after the treatment of MAPK and PI3K inhibitor [31,83], implying NOS and its related signaling pathways are involved in vessel development. It is not unusual for studies to find discrepancy between the biologic effects of P4 and MPA, with Simoncini et al. finding P4-mediated PR promotes the production of NO and endothelial NOS via ERK/Akt phosphorylation, while MPA has an opposite effect. They further verified that P4 and PR antagonist convey no effect on E2-induced NO and endothelial NOS expression, while MPA significantly interferes with E2 actions, suggesting that MPA exerts an inhibitory effect independent of PR [83,84]. A later study confirmed that blocking the glucocorticoid receptor can attenuate the inhibitory effect of MPA [85]. Therefore, progestins might activate nongenomic regulation or crosstalk with other steroid hormone receptors, and thus alter downstream signaling and gene transcription [86,87]. A more recent study confirmed this activation of nongenomic regulation; P4-mediated membrane progestin receptors (mPRs), which can mediate a rapid nongenomic signaling, were found to significantly increase endothelial NOS activity and phosphorylation in HUVECs, accompanied by an activation of the PI3K/Akt and MAPK pathways, ultimately influencing the vascular status and function [88].

### 2.7. Other Downstream Factors

Despite limited evidence, several angiogenic factors are likely to serve a downstream role in progestogen-mediated neovascularization. Platelet-derived growth factor-A (PDGF-A) is known to potentiate VEGF production and release [89], and a paper reported that P4 treatment leads to increased expression of PDGF-A in breast cancer MCF7 and T47D cells [90]. Thrombospondin-1 (TSP-1), an antiangiogenic glycoprotein, is stimulated by P4 in the endometrium, leading to inhibition of the migration of ECs and vessel formation [91]. A consistent conclusion has been made with regard to breast cancer and Ishikawa cells, and progestins have been found to promote TSP-1 expression in a PR-dependent manner [92].

In general, numerous effective factors are involved in progestogen-mediated neovascularization, suggesting that it is a co-regulated process influenced by multiple pertinent angiogenic factors. The conflicting regulation of progestogens on most factors implies their action is probably susceptible to some intrinsic and extrinsic factors.

## 3. Progestogen-Mediated Physiological Neovascularization

P4 is one of the major drivers of physiological changes in the endometrium. Dynamic neovascularization occurs in cyclic changes in the endometrium [93]. The menstrual cycle leads to corresponding changes in P4 concentration, influencing vasculogenesis and angiogenesis in uterus tissue [5,94]. During the early proliferative phase, the serum P4 concentration is relatively low (lower than 1 ng/mL). After ovulation, the endometrium goes into the secretory phase, which is characterized by a high microvascular density, providing a basis for fertilization and implantation, and serum P4 peaks in the mid-secretory phase (10–35 ng/mL) [8,95]. Neovascularization periodically ranges from the proliferative phase to the secretory phase [96,97].

The vasculature growth and VEGF expression exhibits period features corresponding to P4 concentration throughout the menstrual cycle, and a significant increase occurs in the late proliferative and secretory phases, in which P4 levels rapidly reaches their peak [28,98,99]. Meanwhile, increases in EPCs have been observed in peripheral circulation during the secretory phase, and they then migrate to the endometrium [100,101], implying a regulatory correlation between serum P4 and EPC mobilization. Under the influence of ovarian hormones, EPCs acquire an improved angiogenic potential, and P4-mediated PR improves the proliferative ability of EPCs in a dose-dependent manner [102]. Vessel remodeling, a type of endometrial neovascularization characterized by a high average microvessel length and low blood vessel junction, is prominent from the late proliferative to mid-secretory phase [103], accompanied by the proliferation of vascular smooth muscle cells and coverage of mural cells, which contribute to the maturation of endometrial vascular tissues [104,105,106]. A study reported that vessel maturation is primarily related to P4 while E2 has no impact on this process, implying that P4 rather than E2 plays a pivotal role in this process [26]. Later studies confirmed that P4 facilitates vessel development and maturation in the endometrium [107,108]. As a prerequisite in decidual angiogenesis, P4 upregulates VEGF and NOS in the endometrium, and thus elevates blood perfusion and embryo receptivity, contributing to suitable conditions for embryo implantation [25,27,48,109,110,111,112]. Blocking VEGFR-2 can decrease P4-induced decidual angiogenesis during early pregnancy [113]. Meanwhile, embryo implantation and placentation potentiate endometrial angiogenesis by facilitating VEGF generation [114], and a low expression of VEGF in decidual cells and the epithelium of endometrial glands is associated with spontaneous abortion [115]. In a way, the relationship between neovascularization and embryo implantation is based on P4 and its downstream factor. PR knockout renders the uterus unable to undergo decidual transformation even under an artificial decidual stimulus [116]. Blocking P4/PR interferes with the development of capillary vessels in the postovulatory endometrium, leading to the regression of spiral arteries and abnormal veins [117,118]. Although it is a well-established antagonistic feature of P4 to E2-mediated actions [44,119], P4 combined with E2 further enhances human uterine microvascular endothelial cell invasion and sprouting angiogenesis [120].

A significant portion of studies support the pro-angiogenic role of progestins, but several studies indicated that P4 and progestins play an antiangiogenic role in endometrial vascular development. Rashidi et al. demonstrated that P4 attenuates EC proliferation induced by gonadotropins, leading to an inhibitory effect on endometrial angiogenesis [121], and a consistent pattern has been found in ectopic endometrial lesions [68]. Synthetic progestins have a similar influence, and several studies have revealed that the levonorgestrel-releasing subdermal contraceptive has an anti-angiogenic effect, and that it significantly inhibits the proliferation rate of endometrial ECs, which results in reduced endometrial angiogenic activity [122,123]. Further, a sustained peak expression of cyclin E and A has been observed in this process, resulting in arresting of the EC cycle in G1 [124]. Hsu et al. demonstrated that P4-mediated PR can upregulate p21 and p27 in a p53-dependent manner, and that it inhibits DNA synthesis of HUVECs, leading to G0/G1 arrest and a decrease in cell proliferation and capillary-like tube formation [125,126]. This antiangiogenic effect has been observed in the setting of E2 priming, and P4 attenuates E2-induced angiogenesis by inhibiting ER expression, with the PR-A acting as a trans-repressor for ER [13]. A paper reported that P4 attenuates E2-mediated angiogenesis in an ER-independent manner [127], meaning that multiple regulatory patterns are involved in this process.

## 4. Progestogen-Mediated Pathological Neovascularization

Neovascularization conveys a more efficient vascular supply for solid tumors, and it has proven to be an important mechanism supporting tumoral aggressiveness [128]. Progestogens are mainly implicated in the neovascularization of female sex hormone-dependent cancers, including endometrial cancer and breast cancer.

### 4.1. Endometrial Cancer

Compared to the healthy population, lower P4 concentration tends to appear in endometrial cancer patients [129]. P4 inhibits E2-driven growth, and progestogen-mediated PR can prevent endometrial hyperplasia and well-differentiated endometrial cancer from E2-induced growth and invasiveness [130]. Progestin therapy is an effective anti-tumor method for endometrial cancer cases, which can inhibit tumor recurrence and progression in nearly 80% of early stage patients [131]. Publications confirmed the mechanism underlying the inhibitory effect of progestins on endometrial cancer is associated with angiogenesis. After human uterine microvascular ECs were incubated with the conditioned media from Ishikawa cells treated by P4, decreased invasion and tube formation were observed, and these changes were abrogated by blocking PR, implying that the tumor microenvironment functions as a mediator in progestogen actions. Meanwhile, inhibiting Akt signaling could potentiate the antiangiogenic effect [132]. Studies have found that P4 treatment alone cannot alter VEGF in Ishikawa cells [133], but P4, MPA and 17 a-hydroxyprogesterone effectively suppress E2-induced VEGF [134], confirming that progestogens exert antagonistic impacts on E2-induced angiogenesis in endometrial cancer. However, a study reported that progestins exert a synergistic effect with E2 actions. P4 and its metabolite, 17alpha-hydroxyprogesterone, could increase PD-ECGF expression; a fold change in PD-ECGF was observed when it was combined with E2, but this effect could not be induced by MPA, suggesting a discrepant regulation of P4 and progestin on PD-ECGF [72]. Further, a cell-specific regulatory pattern was observed; P4 and MPA suppress E2-induced bFGF secretion in well-differentiated uterine endometrial cancers cells, while they cannot suppress bFGF in poorly differentiated cells [67,69], which accords with the protective effect of progestogens on well-differentiated endometrial cancer.

### 4.2. Breast Cancer

Although serum P4 is generally in the normal range, the activation of its receptors exerts indispensable influence on breast cancer progression [135]. Progestogens that bind to PR promote breast cancer growth. ER upregulates PR expression, and PR is thus a well-validated biomarker for predicting patient response to ER-targeted therapies. However, PR-targeted therapies have failed to progress significantly in clinical therapy for breast cancer, due to confusion regarding the effects of progestogens, which exhibit both stimulatory and inhibitory effects on angiogenesis [9,136]. Hyder et al. first reported that P4 stimulates VEGF expression via PR in human breast cancer cells, and that specificity protein-1 and MAPK signaling are involved in transcriptional and posttranscriptional regulation [49,133,137]. Genomic profile analysis revealed that the expression of VEGF and P4-mediated pathways are both activated in breast cancer [138]. This result is consistent with the results of next-generation sequencing, which have confirmed that VEGF acts as a downstream gene in PR signaling [139]. Later studies confirmed that P4 or synthetic progestins activate angiogenic pathways associated with VEGF, and this effect is preferentially mediated by PR-B and inhibited by blocking PR [140,141,142]. Botelho et al. proposed that P4-mediated VEGF primarily promotes angiogenesis in the early stages of tumorigenesis, which leads to a high aggressiveness [39]. In view of the higher P4 concentrations in young people, this finding can partially explain the more advanced and aggressive nature of breast cancer in adolescents and young adults (age < 40 years) [143]. In addition to VEGF, P4 can induce PDGF-A expression in the breast cancer MCF7 cell [90]. However, a study proposed that PR is not significantly associated with microvessel density in breast cancer [6], while Vameşu et al. confirmed that a significant correlation between high microvessel density and PR negative status exists [144]. P4 and synthetic progestins binding to PR potentiate the expression of anti-angiogenic factor TSP-1 in breast cancer cells [92], implying that P4-PR axis plays an antiangiogenic role in this process. This antiangiogenic effect was validated in a rat model: MPA and fluorinating MPA significantly inhibit angiogenesis, leading to the low growth of mammary carcinomas [145].

In addition, several studies have reported that progestogens influence angiogenesis in other cancer types. A study showed that P4, as well as E2, can stimulate VEGF secretion in non-small cell lung cancer cells [146]. An in vitro study confirmed that P4 and MPA promote A549 to secrete VEGF in order to enhance vascular endothelial cell proliferation, and tumor-associated angiogenesis reaches the maximum when combined with E2 [42]. Similar to its activity in lung cancer, P4-mediated PR has been found to promote VEGF expression in human astrocytoma cell lines, and steroid receptor coactivator-1, which participates in PR downstream gene transcription, exerts a transcriptional modulation in this process [41].

## 5. Discussion

**Figure 3:**
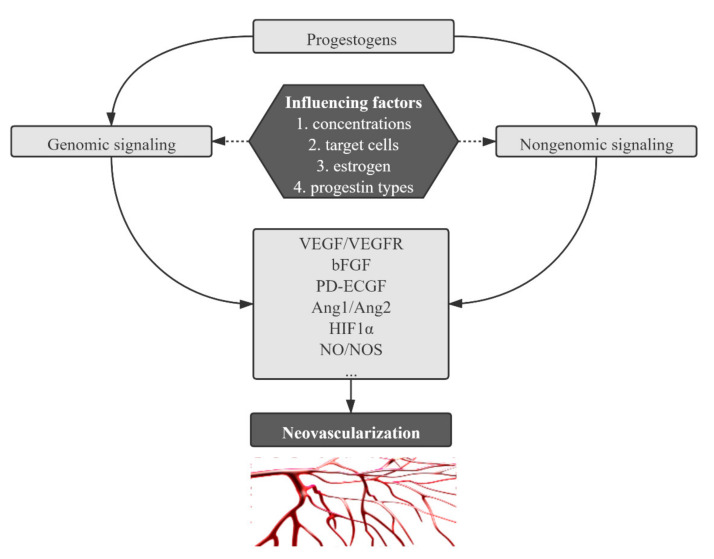
Schematic representation of the main signaling pathways, pertinent angiogenic factors and influencing factors involved in progestogen-mediated neovascularization.

As outlined above, progestogens exert various effects on neovascularization, but conflicting observations in previous studies mean an accurate understanding of their functional characteristics and regulatory patterns remains elusive. We presumed several intrinsic and extrinsic factors that lead to this situation (Figure 3).

First of all, different concentrations of progestogens contribute to contrary outcomes, mainly in in vitro studies. Several studies have found a consistent phenomenon: low concentrations of P4 promote neovascularization, while high concentrations inhibit the original effect [20,35]. The level of PR probably is the cause, and PR expression is influenced by sex hormones; E2 directly regulates the transcription of PR, and P4 exerts a dose-dependent effect on PR expression [20]. The spatiotemporal characteristics of PR expression have been found in endometrial cells; PR rises differently in the epithelium and stroma during peri-implantation, while it is restrictively expressed in stroma cells under changes in ovarian sex hormones after implantation, which means sex hormones can cause dynamic changes in PR [147]. We noted that the anti-angiogenic effect of P4 tends to occur in in vitro studies using a concentration higher than 10^−8^ mol/L (Table 1). Yu et al. confirmed that progestogens exhibit a dose-specific regulation for PR distribution and expression, and that P4 promotes PR expression in low concentrations while it repairs PR expression in high concentrations, which could explain the concentration-dependent influence of progestogens, to a certain extent [20].

Moreover, cell and tissue specificity are other important factors. As shown in Table 1, progestogens boost VEGF expression in diverse breast cancer cell lines, but they exert discrepant effects on different types of human endometrial cells. MPA increases VEGF in epithelial cells, while it reduces VEGF in fibroblast cells [149], implying a cell-specific regulation. Manifold expression patterns of PR isoforms in these cell types could be the root reason for this [150,151], due to the fact that PR isoforms regulate distinct genes profiles. Only 25% of differential expression genes are regulated by both PR-A and PR-B, and PR-mediated responses depend on the coordinated actions of each PR isoform, meaning that different molecular signaling pathways are activated by PR isoforms, which leads to a distinct influence under progestogen treatment [52]. A study confirmed that PR-B prominently enhances VEGF expression, while PR-A inhibits PR-B-mediated actions [142], and thus PR isoforms convey different regulations dependent on the PR-A/PR-B ratio [152]. In addition, conflicting observations are prone to occur in endometrial epithelial cells and stromal cells, and progestogens exert contrary effects even on the same cell type. Heryanto et al. confirmed that there are different proliferative responses to sex hormones among endometrial ECs and epithelial and stromal cells [153]. Further, the procedure of separating epithelial and stromal cells from the normal endometrium could contribute to the suboptimal purity of isolated cells, resulting in an experimental bias. Researchers have proposed that controversial outcomes might be derived from the mistaken identification of endometrial cell types due to technological defects [26]. Regardless, we have noted inconsistent observations between HUVECs and human endometrial endothelial cells [120], suggesting that inevitable cell-specificity occurs even in the well-validated endothelial vascular cell model, and another study confirmed different expression patterns of PR in endothelial cells from different tissues [124]. In contrast to in vitro studies, studies in vivo have reported relatively unanimous observations (Table 2); progestogens promote uterine neovascularization, while they suppress angiogenesis in endometriotic tissues, and this difference can be explained by the patchy expression pattern of PR isoforms in endometriosis [154,155]. In general, tissue and cell specificity make some results controversial.

In addition, E2 priming and its combination with E2 bring changes to original regulatory patterns. Certainly, some amount of the changes come from E2-mediated signaling, which activates cognate nuclear receptor and non-genomic pathways, leading to altered proangiogenic profiles [86]. Further, more than 80% of genes regulated by E2 are regulated by progestogens, while progestogen treatment can alter more than half of E2-induced transcriptomes [14], suggesting that PR dominantly conveys an antagonist to E2-related gene expression and signaling. However, progestogen-mediated PR not only exerts a partial antagonist for E2 [10,157], but a synergy has been observed in many studies (Table 3). One explanation is that progestogen mediates synergistic actions via nongenomic regulation such as mPRα or PGRMC1 signalings, circumventing PR, especially in cell lines with weak expression of PR or without PR [158]. Several studies have reported a positive correlation between PGRMC1 and ER [159,160]. PGRMC1 overexpression promotes E2 biosynthesis and expression of ER-α and its downstream genes [161]. E2 can activate the downstream signaling of PGRMC1, and ER-α knockout downregulates PGRMC1 levels while silencing PGRMC1 inhibits ER-α expression, suggesting a crosstalk and synergistic effect between ER-α and PGRMC1 [162]. An alternative explanation is that E2 potentiates the effect of progestogens, and that even the effect is opposite to its own. Publications have confirmed that E2 upregulates PR, thus enhancing P4′s effect [142,150,163], while the selective PR antagonist inhibits E2-mediated actions, confirming the functional synergy between PR and ER [14]. A publication confirmed that special genes are expressed only under ER and PR coaction, binding to their own sites of promoter regions, and this regulatory mode partially accounts for the synergistic effect [164]. In this regard, the concentration of E2 acts as a contributing factor. Studies have demonstrated that different concentrations of E2 convey contrary influences on the action of progestins [153,165]. Moreover, distinct regulatory modes between E2 and P4 could result in a conflict; P4 tends to exert a late and sustained effect on the expression of VEGF, whereas the effect of E2 is early and transient [20,61,98]. Therefore, observations in different periods can vary, even though they have only been reported in studies on normal cells and tissues. In general, E2 and its receptors make progestogen-mediated neovascularization more complicated [166,167].

Finally, progestogen types can cause conflicts. Due to the different structures among progestogens, altering molecular biofunctions makes natural P4 and synthetic progestins work in individual patterns. Many papers have revealed drug-specific influences: P4 mainly upregulates VEGF in the basal layers of epithelial cells, while the effect of MPA is primarily exerted on stromal and epithelial cells [156]; P4 modulates vascular remodeling by inducing VEGF and endothelial NOS, while the regulation of MPA mainly depends on platelet activation [31,83]. Progestins were proved to have a different regulatory intensity for the same angiogenic factor [148]. Furthermore, the pharmacological effect of PR modulators and P4 antagonists has been reported to work in a cell-specific manner [149]. A study confirmed that P4 and progestins activate or repress unique genomic and proteomic profiles [168], which explains some puzzling findings in previous studies, such as that P4 uniquely affects angiogenesis while MPA affects cell migration [86]. Therefore, some conflicting outcomes are likely due to the use of different progestogen types.

Certainly, the spatial and temporal factors make progestogen-induced actions variable, and thus some observations might deviate from the truth. The question of how to preclude these influencing factors is particularly important for future studies. Progestogens activate the signaling molecules to modulate neovascularization through binding to their receptors, and we noted that ligand-independent activation is widely validated in steroid hormone receptors. In contrast to the classical steroid hormone-dependent manner, ligand-independent activation can be triggered by the steroid hormone receptors themselves, growth factors and various receptors [169,170,171]. The type of activation conveys similar downstream consequences with activation by steroid hormones, and an increased receptor concentration enhances the ligand affinity and potency of post-receptor signaling [172,173]. The P4 receptors, whether they are PR, mPRα or PGRMC1, have been confirmed to be activated in the absence of ligands [58,174,175]. Therefore, gene transfection technology can generate a model with receptor knockdown or overexpression, which provides a feasible and effective method to explore the corresponding change in post-receptor signaling without influences from progestogen concentrations and types. Given the complexity of progestogen-mediated neovascularization, in vitro studies need to be proofed with evidence from in vivo studies. Moreover, there are few direct studies on neovascularization during P4 changes in pathological conditions associated with placenta dysfunction and neuroendocrine neoplasms, and this is a direction worthy of investigation. Furthermore, future studies can explore the role of progestogens in subdivisions of neovascularization, such as sprouts, intussusception and elongation, which might provide novel insight into the mechanisms of progestogen actions.

## Figures and Tables

**Table 1:** The influence of progestogens in in vitro studies: ↑ indicates a promoting effect; ↓ indicates an inhibitory effect. E2: estradiol; LNG: levonorgestrel; MPA: medroxyprogesterone acetate; NET: norethindrone; P4: progesterone; PD-ECGF: platelet-derived endothelial cell growth factor; PR: progesterone receptor; tPA: tissue plasminogen activator; VEGF: vascular endothelial growth factor; VEGFR: vascular endothelial growth factor receptor.

Reference	Progestogens	Object	Effect	Concentration
[133]	P4	Human breast cancer cells T47-D	VEGF ↑	10^−8^ mol/L
[39]	P4	Human breast cancer cells MCF7	VEGF ↑	-
[141]	P4 MPA NET Norgestrel	Human breast cancer cells T47-D	VEGF ↑	10^−8^ mol/L
[49]	P4	Human breast cancer cells T47-D	VEGF ↑	10^−8^ mol/L
[142]	P4 MPA MGA	Human breast cancer cells T47-D	VEGF ↑ (PR-B cell) P4-PR-B-induced VEGF ↓ (PR-A cell)	10^−8^ mol/L
[90]	P4	Human breast cancer cells MCF7	Platelet-derived growth factor A ↑ VEGF ↑	10^−8^ mol/L
[137]	P4 MPA	Human breast cancer cells BT-474 and T47-D	VEGF ↑	10^−8^ mol/L
[92]	P4 MPA MGA	Human breast cancer cells T47-D and BT-474	Thrombospondin-1 ↑	P4: 10^−9^ mol/L M MPA: 10^−8^ mol/L MGA: 10^−8^ mol/L
[63]	P4 MPA	Human breast cancer cells T47-D, BT-474, HCC-1428 and MDAMB-231 Human umbilical vein endothelial cells	VEGF ↑	P4: 10^−8^ mol/L MPA: 10^−8^ mol/L
[133]	MPA Norgestrel NET, Norethynodrel	Human breast cancer cells T47-D	VEGF ↑	MPA: 10^−8^ mol/L Norgestrel: 10^−8^ mol/L NET: 10^−8^ mol/L Norethynodrel: 10^−8^ mol/L
[57]	Membrane-impermeable P4 conjugate	Human breast cancer cells MCF7	VEGF mRNA↑	10^−6^ mol/L
[72]	P4 MPA	Human Ishikawa cells	PD-ECGF ↑	10^−8^ mol/L
[67,69]	P4	Human Ishikawa cells	Basic fibroblast growth factor ↓	10^−8^ mol/L
[148]	P4 MPA LNG NET	Human Ishikawa cells	Thrombospondin-1 ↑	10^−8^ to 10^−6^ mol/L
[109]	P4	Human Ishikawa cells Human endometrial epithelial cells	VEGF ↑	10^−8^ mol/L
[71]	P4	Human endometrial epithelial cells Human endometrial stromal cells	PD-ECGF ↑ (stromal cells P4 treatment) PD-ECGF ↑ (epithelial cells under P4+ TGFβ1 treatment)	P4: 5 × 10^−8^ mol/L TGFβ1: 10 ng/mL
[55]	P4	Human endometrial endothelial cells	Angiogenic capacity and vascular tube formation ↑	10^−10^ mol/L
[74]	P4	Human uterine microvascular endothelial cells	Angiopoietin-2 ↑	10^−5^ mol/L
[132]	R5020	Human uterine microvascular endothelial cells incubated with conditioned media from Ishikawa cells treated by R5020	Invasion ↓	10^−8^ mol/L
[91]	P4	Human endometrial stromal cells	Thrombospondin-1 ↑	10^−8^ to 5×10^−8^ mol/L
[75]	MPA	Human endometrial stromal cells	Angiopoietin-1 ↑	10^−7^ mol/L
[53]	P4 Dienogest	Human endometriotic epithelial cells	VEGF ↓	P4: 10^−7^ mol/L Dienogest: 10^−7^ mol/L
[44]	P4 MPA NET LNG DNG	Human endometrial stromal cells	VEGF ↓	P4: 10^−7^ mol/L MPA: 10^−9^ to 10^−7^ mol/L NET: 10^−9^ to 10^−7^ mol/L LNG: 10^−9^ to 10^−7^ mol/L DNG: 10^−9^ to 10^−7^ mol/L
[149]	MPA	Human endometrial epithelial cells Human endometrial fibroblasts	VEGF ↓ (fibroblast) VEGF ↑ (epithelial cell)	10^−6^ mol/L
[98]	MPA	Human endometrial stomal cells	VEGF ↑	10^−7^ mol/L
[86]	LNG NETA	Human endometrial stromal fibroblasts	VEGF ↓	LNG: 5.47×10^−7^ mol/L NETA: 0.29×10^−6^ mol/L
[54]	P4	Human umbilical vein endothelial cells	Migration ↓	0.5 ×10^−8^ to 10^−6^ mol/L
[125,126]	P4	Human umbilical vein endothelial cells	G0/G1 arrest	0.5 ×10^−6^ mol/L
[82,88]	P4	Human umbilical vein endothelial cells	Endothelial nitric oxide synthase ↑	2 ×10^−8^ mol/L
[124]	P4	Human dermal endothelial cells	Arrest endothelial cell cycle in G1	0.5 × 10^−8^ mol/L
[46]	P4	Immortalized mouse brain endothelial cells	tPA-induced VEGF↓	P4: 2× 10^−7^ mol/L (tPA: 20 μg/mL)
[102]	P4	Human endothelial progenitor cells	Proliferation ↑	10^−7^ mol/L
[20,51]	P4	Endothelial progenitor cells (rats)	Angiogenic potential ↑ (10^−9^ mol/L) Angiogenic potential ↓ (10^−7^ mol/L)	10^−7^to 10^−9^ mol/L
[31,83]	P4 MPA	Endothelial cells (rats)	P4-induced VEGF ↑ P4-induced nitric oxide synthase ↑ P4 and MPA promote capillary-like tube formation↑	P4: 10^−9^ to 10^−7^ mol/L MPA: 10^−9^ to 10^−7^ mol/L
[84]	P4 MPA	Human endothelial cells	P4-induced endothelial nitric oxide synthase ↑ MPA-induced endothelial nitric oxide synthase ↓	P4: 10^−8^ mol/L MPA: 10^−8^ mol/L
[36]	P4	Granulosa cells from large follicles	VEGFA mRNA ↑	300 ng/mL (9.5×10^−7^ mol/L)
[35]	P4	Ovary bovine granulosa cells (cows)	VEGF120 ↑ (10ng/mL) HIF1α ↑ (10ng/mL) VEGFR2 ↑ (10ng/mL) VEGF120 ↓ (100ng/mL) HIF1α ↓ (100ng/mL) VEGFR2 ↓ (100ng/mL)	10 ng/mL (3.18 ×10^−8^ mol/L) 100 ng/mL (3.18×10^−7^ mol/L)
[42]	P4 MPA	Human lung cancer A549	VEGF ↑	P4: 10^−8^ mol/L MPA: 10^−8^ mol/L
[41]	P4	Human astrocytoma D54 cell line	VEGF ↑	10^−8^ mol/L
[37]	P4	Human fibroblasts and alveolar cells type II (mice)	VEGF ↑ (no significance)	10^–8^ mol/L
[38]	P4	Human pyogenic granuloma (during pregnancy) Human monocyte-like U937 cells	VEGF ↑ Basic fibroblast growth factor ↑	100ng/mL (plus 5mg/mL LPS)
[56]	Membrane-impermeable P4 conjugate	Retinal glial cells (porcine)	VEGF ↑	10^−7^ mol/L

**Table 2:** The influence of progestogens in in vivo studies: ↑ indicates a promoting effect; ↓ indicates an inhibitory effect. FMPA: 9alpha-fluoromedoroxyprogesterone acetate.

Reference	Treatment	Object	Effect
[26]	P4 Estrogen priming + P4	Endometrium endothelial cell (mice)	P4 promoting VEGF Estrogen priming inhibits P4-induced angiogenesis
[141]	MPA	Mammary tumors (rats)	CD34-positive blood vessels ↑
[27]	P4	Uterine (mice)	VEGF ↑ VEGFR1 ↓ VEGFR2 ↓
[61]	P4 ↓P4 + E2	Uterine (mice)	VEGF ↑ and VEGFR2 ↑ (P4 treatment) ↓VEGF ↑ (P4+E2 treatment)
[132]	P4	Endometrial cancer (mice)	Angiogenesis ↓
[33]	P4	Ovarian tissue (mice)	VEGF ↑
[48]	P4	Uterus (mice)	VEGFA ↑ VEGFR2 ↑
[153]	P4 + E2	Endometrial endothelial cells (male mice)	Epithelial and endothelial cell proliferation ↓ (E2 10ng + P4) Endothelial cell proliferation ↑ (E2 100ng + P4)
[121]	P4	Endometrium (mice)	Angiogenesis ↓
[107]	P4	Endometrium (mice)	Endometrial vascular maturation ↑
[108]	P4	Endometrium (pig)	Angiogenesis ↑
[68]	P4 Dydrogesterone Dihydrodydrogesterone	Human ectopic endometrial lesions (mice)	Basic fibroblast growth factor ↓ (P4 or dydrogesterone treatment) VEGF ↓ (dydrogesterone or dihydrodydrogesterone treatment)
[154]	P4	Endometriosis (mice)	Angiogenesis ↓
[119]	P4	Ectopic uterine tissue (mice)	The size of ectopic uterine tissues ↓ (P4 treatment) The size of ectopic uterine tissues ↑ (E2 treatment) P4 inhibits E2 effects
[64]	P4	Endometrium endometrial stroma (macaques)	VEGF ↓ in stroma of the superficial endometrial zones
[46]	tPA + P4	Brain tissue (rats)	Inhibiting tPA-induced VEGF
[81]	P4 P4 + E2	Uterine artery endothelium (sheep)	Endothelial nitric oxide synthase ↑ (P4 or P4 + E2 treatment)
[145]	MPA FMPA	Corneal assays (rabbits)	Angiogenesis ↓
[38]	P4 P4 + E2	Air ponch granuloma (mice)	VEGF ↑ (P4 or P4 + E2 treatment)
[45]	P4	Diabetic nephropathy model (rats)	VEGF ↓
[32]	P4	Skin flap (rats)	VEGF ↑
[127]	P4	Bladder detrusor (rats)	Blood vessel density ↓
[124]	P4	Re-endothelialization assays (rats)	Re-endothelialization of injured aortae ↓
[47]	P4	Brains (rats)	VEGF ↓ No change in microvessel density
[20,50]	P4	Brains (rats)	Circulating endothelial pro-genitor cells ↑ Vessel density ↑
[29,30]	P4	Uterine (rats)	VEGF ↑ Endothelial cell density ↑ miR-16-5p ↑ miR-17-5p ↑
[78]	P4	Uterus (ovines)	VEGFA ↑ HIF1A ↑ HIF2A ↑
[140]	P4 MPA	Mammary glands (mice)	Average number of small sized blood vessels ↑ (MPA treatment, but not P4)
[156]	P4 MPA	Mammary tumor (rats)	VEGF ↑ in basal cell (P4 treatment) VEGF ↑ in epithelial and stromal cells (MPA treatment)

**Table 3:** The combined action of progestogens and E2; ↑ indicates a promoting effect; ↓ indicates an inhibitory effect.

Reference	Treatment	Object	Effect	Concentration
[134]	E2 priming + P4 E2 priming + MPA E2 priming + 17a-hydroxyprogesterone	Human Ishikawa cells	Inhibiting E2-induced VEGF	E2: 10^−8^ mol/L P4: 10^−6^ mol/L MPA: 10^−6^ mol/L 17a-hydroxyprogesterone: 10^−6^ mol/L
[67,69]	E2 priming + P4 E2 priming + MPA E2 priming +17a-hydroxyprogesterone	Human Ishikawa cells	Inhibiting E2-induced basic fibroblast growth factor	E2: 10^−8^ mol/L P4: 10^−8^ mol/L MPA: 10^−6^ mol/L 17a-hydroxyprogesterone: 10^−6^ mol/L
[72]	E2 priming + P4 E2 priming + MPA E2 priming + 17a-hydroxyprogesterone	Human Ishikawa cells	Promoting E2-induced PD-ECGF (E2 priming + P4, E2 priming + 17a-hydroxyprogesterone, but not E2 priming +MPA)	E2: 10^−8^ mol/L P4: 10^−8^ mol/L MPA: 10^−6^ mol/L 17a-hydroxyprogesterone: 10^−6^ mol/L
[142]	P4 + E2	Human breast cancer cells T47-D	VEGF ↑ (PR-B cells) No effect on VEGF expression (PR-A cells)	P4: 10^−8^ mol/L E2: 10^−6^ mol/L
[35]	P4 + E2	Ovary granulosa cells(cows)	VEGF120 ↓ VEGFR2 ↓	P4: 10 ng/mL E2: 1 ng/mL
[37]	P4 + E2	Lung fibroblasts and alveolar cells type II (mice)	VEGF ↑	E2: 10^–8^ to 10^–6^ mol/L P4: 10^–8^ to 10^–6^ mol/L
[38]	P4 + E2	Human monocyte-like U937 cells	VEGF ↑	E2: 20ng/mL P4: 100ng/mL (pretreated by LPS 5mg/mL)
[42]	P4 + E2 E2 priming + MPA	Human lung cancer A549	VEGF ↑ Promoting E2-induced VEGF	P4: 10^−8^ mol/L MPA: 10^−8^ mol/L E2: 10^−8^ mol/L
[86]	P4 + E2 LNG + E2 NETA + E2	Human endometrial stromal fibroblasts	VEGFA mRNA ↓	P4: 10^−6^ mol/L LNG: 5.47×10^−7^ mol/L NETA: 0.294×10^−6^ mol/L E2: 10^−8^ mol/L
[25]	P4 + E2	Human endometrial stromal cells	VEGF189 mRNA ↑	E2: 10^−8^ mol/L P4: 10^−6^ mol/L (pretreated by EGF 20 ng/mL)
[98]	E2 priming +MPA	Human endometrial stromal cells	Promoting E2-induced VEGF	E2: 10^−8^ mol/L MPA: 10^−7^ mol/L
[44]	E2 priming + P4 E2 priming + MPA E2 priming + NET E2 priming + LNG E2 priming + dienogest	Human endometrial stromal cells	Inhibiting E2-induced VEGF	P4: 10^−7^ mol/L MPA: 10^−9^ to 10^−7^ mol/L NET: 10^−9^ to 10^−7^ mol/L LNG: 10^−9^ to 10^−7^ mol/L DNG: 10^−9^ to 10^−7^ mol/L
[28]	MPA + E2	Human endometrial stromal cells	Thrombin ↑ VEGF ↑	E2: 10^−8^ mol/L MPA: 10^−7^ mol/L
[24]	E2 priming + MPA	Human endometrial stromal cells	Promoting E2-induced VEGF	P4: 10^−7^ mol/L E2: 10^−8^ mol/L
[149]	E2 priming + MPA	Human endometrial epithelial cells Human endometrial fibroblasts	Inhibiting E2-induced VEGF (fibroblasts) Promoting E2-induced VEGF (epithelial)	MPA: 10^−6^ mol/L E2: 10^−8^ mol/L
[120]	P4 + E2	Human uterine microvascular endothelial cells	Invasion ↑	E2: 10^−10^ to 10^−8^ mol/L P4: 10^−8^ to 10^−6^ mol/L
[21]	MPA + E2	Endothelial progenitor cells (human)	Partially inhibiting E2-induced proliferation (MPA < 10^−5^ mol/L) Significantly inhibiting E2-induced proliferation (MPA > 10^−4^ mol/L)	E2: 10^−8^ mol/L MPA: 10^−5^ to 10^−4^ mol/L
[55]	P4 + E2	Human endometrial endothelial cell	Proliferation ↑	E2: 10^−8^ mol/L P4: 10^−8^ mol/L

## References

[1] Ribatti D., Pezzella F. (2021). Overview on the Different Patterns of Tumor Vascularization. Cells.

[2] Demir R., Yaba A., Huppertz B. (2010). Vasculogenesis and angiogenesis in the endometrium during menstrual cycle and implantation. Acta Histochem..

[3] Balaji S., King A., Crombleholme T.M., Keswani S.G. (2013). The Role of Endothelial Progenitor Cells in Postnatal Vasculogenesis: Implications for Therapeutic Neovascularization and Wound Healing. Adv. Wound Care.

[4] Brown J.M. (2014). Vasculogenesis: A crucial player in the resistance of solid tumours to radiotherapy. Br. J. Radiol..

[5] Perrot-Applanat M., Ancelin M., Buteau-Lozano H., Meduri G., Bausero P. (2000). Ovarian steroids in endometrial angiogenesis. Steroids.

[6] Bharti J.N., Rani P., Kamal V., Agarwal P.N. (2015). Angiogenesis in Breast Cancer and its Correlation with Estrogen, Progesterone Receptors and other Prognostic Factors. J. Clin. Diagn. Res..

[7] Zheng Z.Y., Bay B.H., Aw S.E., Lin V.C. (2005). A novel antiestrogenic mechanism in progesterone receptor-transfected breast cancer cells. J. Biol. Chem..

[8] Taraborrelli S. (2015). Physiology, production and action of progesterone. Acta Obstet. Gynecol. Scand..

[9] Singhal H., Greene M.E., Zarnke A.L., Laine M., Al Abosy R., Chang Y.F., Dembo A.G., Schoenfelt K., Vadhi R., Qiu X. (2018). Progesterone receptor isoforms, agonists and antagonists differentially reprogram estrogen signaling. Oncotarget.

[10] Mohammed H., Russell I.A., Stark R., Rueda O.M., Hickey T.E., Tarulli G.A., Serandour A.A., Birrell S.N., Bruna A., Saadi A. (2015). Progesterone receptor modulates ERα action in breast cancer. Nature.

[11] Daniel A.R., Gaviglio A.L., Knutson T.P., Ostrander J.H., D’Assoro A.B., Ravindranathan P., Peng Y., Raj G.V., Yee D., Lange C.A. (2015). Progesterone receptor-B enhances estrogen responsiveness of breast cancer cells via scaffolding PELP1- and estrogen receptor-containing transcription complexes. Oncogene.

[12] Orshal J.M., Khalil R.A. (2004). Gender, sex hormones, and vascular tone. Am. J. Physiol. Regul. Integr. Comp. Physiol..

[13] Edwards D.P. (2005). Regulation of signal transduction pathways by estrogen and progesterone. Annu. Rev. Physiol..

[14] Singhal H., Greene M.E., Tarulli G., Zarnke A.L., Bourgo R.J., Laine M., Chang Y.F., Ma S., Dembo A.G., Raj G.V. (2016). Genomic agonism and phenotypic antagonism between estrogen and progesterone receptors in breast cancer. Sci. Adv..

[15] Horwitz K.B., Sartorius C.A. (2020). 90 YEARS OF PROGESTERONE: Progesterone and progesterone receptors in breast cancer: Past, present, future. J. Mol. Endocrinol..

[16] Trenti A., Tedesco S., Boscaro C., Trevisi L., Bolego C., Cignarella A. (2018). Estrogen, Angiogenesis, Immunity and Cell Metabolism: Solving the Puzzle. Int. J. Mol. Sci..

[17] Mints M., Jansson M., Sadeghi B., Westgren M., Uzunel M., Hassan M., Palmblad J. (2008). Endometrial endothelial cells are derived from donor stem cells in a bone marrow transplant recipient. Hum. Reprod..

[18] Bikfalvi A. (2007). Angiogenesis: Molecular mechanisms of activation, promotion and maintenance. Off. J. Balk. Union Oncol..

[19] Krenning G., van Luyn M.J., Harmsen M.C. (2009). Endothelial progenitor cell-based neovascularization: Implications for therapy. Trends Mol. Med..

[20] Yu P., Li S., Zhang Z., Wen X., Quan W., Tian Q., Gao C., Su W., Zhang J., Jiang R. (2017). Progesterone-mediated angiogenic activity of endothelial progenitor cell and angiogenesis in traumatic brain injury rats were antagonized by progesterone receptor antagonist. Cell Prolif..

[21] Liu L.H., Lai Y., Linghu L.J., Liu Y.F., Zhang Y. (2015). Effect of different concentrations of medroxy-progesterone acetate combined with 17β-estradiol on endothelial progenitor cells. Eur. Rev. Med. Pharmacol. Sci..

[22] Ren R., Guo J., Shi J., Tian Y., Li M., Kang H. (2020). PKM2 regulates angiogenesis of VR-EPCs through modulating glycolysis, mitochondrial fission, and fusion. J. Cell Physiol..

[23] Shweiki D., Itin A., Neufeld G., Gitay-Goren H., Keshet E. (1993). Patterns of expression of vascular endothelial growth factor (VEGF) and VEGF receptors in mice suggest a role in hormonally regulated angiogenesis. J. Clin. Investig..

[24] Lebovic D.I., Shifren J.L., Ryan I.P., Mueller M.D., Korn A.P., Darney P.D., Taylor R.N. (2000). Ovarian steroid and cytokine modulation of human endometrial angiogenesis. Hum. Reprod..

[25] Ancelin M., Buteau-Lozano H., Meduri G., Osborne-Pellegrin M., Sordello S., Plouët J., Perrot-Applanat M. (2002). A dynamic shift of VEGF isoforms with a transient and selective progesterone-induced expression of VEGF189 regulates angiogenesis and vascular permeability in human uterus. Proc. Natl. Acad. Sci. USA.

[26] Walter L.M., Rogers P.A., Girling J.E. (2005). The role of progesterone in endometrial angiogenesis in pregnant and ovariectomised mice. Reproduction.

[27] Boroujeni M.B., Boroujeni N.B., Gholami M. (2016). The effect of progesterone treatment after ovarian induction on endometrial VEGF gene expression and its receptors in mice at pre-implantation time. Iran. J. Basic Med. Sci..

[28] Lockwood C.J., Krikun G., Koo A.B., Kadner S., Schatz F. (2002). Differential effects of thrombin and hypoxia on endometrial stromal and glandular epithelial cell vascular endothelial growth factor expression. J. Clin. Endocrinol. Metab..

[29] Salmasi S., Sharifi M., Rashidi B. (2021). Ovarian stimulation and exogenous progesterone affect the endometrial miR-16-5p, VEGF protein expression, and angiogenesis. Microvasc. Res..

[30] Salmasi S., Sharifi M., Rashidi B. (2021). Evaluating the effect of ovarian stimulation and exogenous progesterone on CD31-positive cell density, VEGF protein, and miR-17-5p expression of endometrium immediately before implantation. Biomed. Pharmacother. Biomed. Pharmacother..

[31] Cutini P.H., Massheimer V.L. (2019). In vitro effects of progesterone and the synthetic progestin medroxyprogesterone acetate on vascular remodeling. Mol. Cell Endocrinol..

[32] Dingsheng L., Zengbing L., Dong H. (2016). Favorable effects of progesterone on skin random flap survival in rats. Iran. J. Basic Med. Sci..

[33] Narimani L., Boroujeni N.B., Gholami M., Anbari K., Alavi S.E.R., Ahmadi S.A.Y., Boroujeni M.B. (2020). Pre-Implantation Effects of Progesterone Administration on Ovarian Angiogenesis after Ovarian Stimulation: A Histological, Hormonal, and Molecular Analysis. JBRA Assist. Reprod..

[34] Christensen A.C., Haresign W., Khalid M. (2014). Progesterone exposure of seasonally anoestrous ewes alters the expression of angiogenic growth factors in preovulatory follicles. Theriogenology.

[35] Shimizu T., Miyamoto A. (2007). Progesterone induces the expression of vascular endothelial growth factor (VEGF) 120 and Flk-1, its receptor, in bovine granulosa cells. Anim. Reprod. Sci..

[36] Nichols J.A., Perego M.C., Schütz L.F., Hemple A.M., Spicer L.J. (2019). Hormonal regulation of vascular endothelial growth factor A (VEGFA) gene expression in granulosa and theca cells of cattle1. J. Anim. Sci..

[37] Trotter A., Kipp M., Schrader R.M., Beyer C. (2009). Combined application of 17beta-estradiol and progesterone enhance vascular endothelial growth factor and surfactant protein expression in cultured embryonic lung cells of mice. Int. J. Pediatr..

[38] Yuan K., Wing L.C., Lin M.T. (2002). Pathogenetic Roles of Angiogenic Factors in Pyogenic Granulornas in Pregnancy Are Modulated by Female Sex Hormones. J. Periodontol..

[39] Botelho M.C., Soares R., Alves H. (2015). Progesterone in Breast Cancer Angiogenesis. SM J. Reprod. Health Infertil..

[40] Fujimoto J., Toyoki H., Jahan I., Alam S.M., Sakaguchi H., Sato E., Tamaya T. (2005). Sex steroid-dependent angiogenesis in uterine endometrial cancers. J. Steroid Biochem. Mol. Biol..

[41] Hernández-Hernández O.T., González-García T.K., Camacho-Arroyo I. (2012). Progesterone receptor and SRC-1 participate in the regulation of VEGF, EGFR and Cyclin D1 expression in human astrocytoma cell lines. J. Steroid Biochem. Mol. Biol..

[42] Marquez-Garban D.C., Mah V., Alavi M., Maresh E.L., Chen H.W., Bagryanova L., Horvath S., Chia D., Garon E., Goodglick L. (2011). Progesterone and estrogen receptor expression and activity in human non-small cell lung cancer. Steroids.

[43] Keck C., Herchenbach D., Pfisterer J., Breckwoldt M. (1998). Effects of 17beta-estradiol and progesterone on interleukin-6 production and proliferation of human umbilical vein endothelial cells. Exp. Clin. Endocrinol. Diabetes.

[44] Okada H., Okamoto R., Tsuzuki T., Tsuji S., Yasuda K., Kanzaki H. (2011). Progestins inhibit estradiol-induced vascular endothelial growth factor and stromal cell-derived factor 1 in human endometrial stromal cells. Fertil. Steril..

[45] Al-Trad B., Ashankyty I.M., Alaraj M. (2015). Progesterone ameliorates diabetic nephropathy in streptozotocin-induced diabetic Rats. Diabetol. Metab. Syndr..

[46] Won S., Lee J.H., Wali B., Stein D.G., Sayeed I. (2014). Progesterone attenuates hemorrhagic transformation after delayed tPA treatment in an experimental model of stroke in rats: Involvement of the VEGF-MMP pathway. J. Cereb. Blood Flow Metab. Off. J. Int. Soc. Cereb. Blood Flow Metab..

[47] Jiang C., Zuo F., Wang Y., Lu H., Yang Q., Wang J. (2017). Progesterone Changes VEGF and BDNF Expression and Promotes Neurogenesis After Ischemic Stroke. Mol. Neurobiol..

[48] Kim M., Park H.J., Seol J.W., Jang J.Y., Cho Y.S., Kim K.R., Choi Y., Lydon J.P., Demayo F.J., Shibuya M. (2013). VEGF-A regulated by progesterone governs uterine angiogenesis and vascular remodelling during pregnancy. EMBO Mol. Med..

[49] Hyder S.M., Chiappetta C., Stancel G.M. (2001). Pharmacological and endogenous progestins induce vascular endothelial growth factor expression in human breast cancer cells. Int. J. Cancer.

[50] Li Z., Wang B., Kan Z., Zhang B., Yang Z., Chen J., Wang D., Wei H., Zhang J.N., Jiang R. (2012). Progesterone increases circulating endothelial progenitor cells and induces neural regeneration after traumatic brain injury in aged rats. J. Neurotrauma.

[51] Yu P., Zhang Z., Li S., Wen X., Quan W., Tian Q., Chen J., Zhang J., Jiang R. (2016). Progesterone modulates endothelial progenitor cell (EPC) viability through the CXCL12/CXCR4/PI3K/Akt signalling pathway. Cell Prolif..

[52] Kaya H.S., Hantak A.M., Stubbs L.J., Taylor R.N., Bagchi I.C., Bagchi M.K. (2015). Roles of progesterone receptor A and B isoforms during human endometrial decidualization. Mol. Endocrinol..

[53] Ichioka M., Mita S., Shimizu Y., Imada K., Kiyono T., Bono Y., Kyo S. (2015). Dienogest, a synthetic progestin, down-regulates expression of CYP19A1 and inflammatory and neuroangiogenesis factors through progesterone receptor isoforms A and B in endometriotic cells. J. Steroid Biochem. Mol. Biol..

[54] Lee T.S., Lin J.J., Huo Y.N., Lee W.S. (2015). Progesterone Inhibits Endothelial Cell Migration Through Suppression of the Rho Activity Mediated by cSrc Activation. J. Cell. Biochem..

[55] Kayisli U.A., Luk J., Guzeloglu-Kayisli O., Seval Y., Demir R., Arici A. (2004). Regulation of angiogenic activity of human endometrial endothelial cells in culture by ovarian steroids. J. Clin. Endocrinol. Metab..

[56] Swiatek-De Lange M., Stampfl A., Hauck S.M., Zischka H., Gloeckner C.J., Deeg C.A., Ueffing M. (2007). Membrane-initiated effects of progesterone on calcium dependent signaling and activation of VEGF gene expression in retinal glial cells. Glia.

[57] Neubauer H., Adam G., Seeger H., Mueck A.O., Solomayer E., Wallwiener D., Cahill M.A., Fehm T. (2009). Membrane-initiated effects of progesterone on proliferation and activation of VEGF in breast cancer cells. Climacteric J. Int. Menopause Soc..

[58] Peluso J.J., Liu X., Uliasz T., Pru C.A., Kelp N.C., Pru J. (2018). PGRMC1/2 promotes luteal vascularization and maintains the primordial follicles of mice. Reproduction.

[59] Andrikopoulou M., Chatzistamou I., Gkilas H., Vilaras G., Sklavounou A. (2013). Assessment of angiogenic markers and female sex hormone receptors in pregnancy tumor of the gingiva. J. Oral Maxillofac. Surg. Off. J. Am. Assoc. Oral Maxillofac. Surg..

[60] Meduri G., Bausero P., Perrot-Applanat M. (2000). Expression of vascular endothelial growth factor receptors in the human endometrium: Modulation during the menstrual cycle. Biol. Reprod..

[61] Ma W., Tan J., Matsumoto H., Robert B., Abrahamson D.R., Das S.K., Dey S.K. (2001). Adult tissue angiogenesis: Evidence for negative regulation by estrogen in the uterus. Mol. Endocrinol..

[62] Das D., Saikia P.J., Gowala U., Sarma H.N. (2021). Cell Specific Expression of Vascular Endothelial Growth Factor Receptor-2 (Flk-1/KDR) in Developing Mice Embryo and Supporting Maternal Uterine Tissue during Early Gestation (D4-D7). Int. J. Fertil. Steril..

[63] Liang Y., Hyder S.M. (2005). Proliferation of endothelial and tumor epithelial cells by progestin-induced vascular endothelial growth factor from human breast cancer cells: Paracrine and autocrine effects. Endocrinology.

[64] Nayak N.R., Brenner R.M. (2002). Vascular proliferation and vascular endothelial growth factor expression in the rhesus macaque endometrium. J. Clin. Endocrinol. Metab..

[65] Nayak N.R., Critchley H.O., Slayden O.D., Menrad A., Chwalisz K., Baird D.T., Brenner R.M. (2000). Progesterone withdrawal up-regulates vascular endothelial growth factor receptor type 2 in the superficial zone stroma of the human and macaque endometrium: Potential relevance to menstruation. J. Clin. Endocrinol. Metab..

[66] Katoh M. (2016). Therapeutics Targeting FGF Signaling Network in Human Diseases. Trends Pharmacol. Sci..

[67] Fujimoto J., Hori M., Ichigo S., Hirose R., Sakaguchi H., Tamaya T. (1997). Plausible novel therapeutic strategy of uterine endometrial cancer with reduction of basic fibroblast growth factor secretion by progestin and O-(chloroacetyl-carbamoyl) fumagillol (TNP-470; AGM-1470). Cancer Lett..

[68] Mönckedieck V., Sannecke C., Husen B., Kumbartski M., Kimmig R., Tötsch M., Winterhager E., Grümmer R. (2009). Progestins inhibit expression of MMPs and of angiogenic factors in human ectopic endometrial lesions in a mouse model. Mol. Hum. Reprod..

[69] Fujimoto J., Hori M., Ichigo S., Hirose R., Tamaya T. (1997). Antiestrogenic compounds inhibit estrogen-induced expressions of basic fibroblast growth factor and its mRNA in well-differentiated endometrial cancer cells. Gen. Pharmacol..

[70] Fujimoto J., Ichigo S., Sakaguchi H., Hirose R., Tamaya T. (1998). Expression of platelet-derived endothelial cell growth factor and its mRNA in uterine endometrium during the menstrual cycle. Mol. Hum. Reprod..

[71] Zhang L., Mackenzie I.Z., Rees M.C., Bicknell R. (1997). Regulation of the expression of the angiogenic enzyme platelet-derived endothelial cell growth factor/thymidine phosphorylase in endometrial isolates by ovarian steroids and cytokines. Endocrinology.

[72] Aoki I., Fujimoto J., Tamaya T. (2003). Effects of various steroids on platelet-derived endothelial cell growth factor (PD-ECGF) and its mRNA expression in uterine endometrial cancer cells. J. Steroid Biochem. Mol. Biol..

[73] Zavarhei M.D., Bidgoli S.A., Ziyarani M.M., Shariatpanahi M., Ardalan F.A. (2007). Progesterone receptor positive colorectal tumors have lower thymidine phosphorylase expression: An immunohistochemical study. Pak. J. Biol. Sci..

[74] Park Y.G., Choi J., Seol J.W. (2020). Angiopoietin-2 regulated by progesterone induces uterine vascular remodeling during pregnancy. Mol. Med. Rep..

[75] Krikun G., Critchley H., Schatz F., Wan L., Caze R., Baergen R.N., Lockwood C.J. (2002). Abnormal uterine bleeding during progestin-only contraception may result from free radical-induced alterations in angiopoietin expression. Am. J. Pathol..

[76] Hickey M., Krikun G., Kodaman P., Schatz F., Carati C., Lockwood C.J. (2006). Long-term progestin-only contraceptives result in reduced endometrial blood flow and oxidative stress. J. Clin. Endocrinol. Metab..

[77] Pan X.Y., Zhang Z.H., Wu L.X., Wang Z.C. (2015). Effect of HIF-1a/VEGF signaling pathway on plasma progesterone and ovarian prostaglandin F_2_a secretion during luteal development of pseudopregnant rats. Genet. Mol. Res..

[78] Song G., Kim J., Bazer F.W., Spencer T.E. (2008). Progesterone and interferon tau regulate hypoxia-inducible factors in the endometrium of the ovine uterus. Endocrinology.

[79] Cattaneo M.G., Vanetti C., Decimo I., Di Chio M., Martano G., Garrone G., Bifari F., Vicentini L.M. (2017). Sex-specific eNOS activity and function in human endothelial cells. Sci. Rep..

[80] Duda D.G., Fukumura D., Jain R.K. (2004). Role of eNOS in neovascularization: NO for endothelial progenitor cells. Trends Mol. Med..

[81] Rupnow H.L., Phernetton T.M., Shaw C.E., Modrick M.L., Bird I.M., Magness R.R. (2001). Endothelial vasodilator production by uterine and systemic arteries. VII. Estrogen and progesterone effects on eNOS. Am. J. Physiol. Heart Circ. Physiol..

[82] You Y., Tan W., Guo Y., Luo M., Shang F.F., Xia Y., Luo S. (2020). Progesterone promotes endothelial nitric oxide synthase expression through enhancing nuclear progesterone receptor-SP-1 formation. Am. J. Physiol. Heart Circ. Physiol..

[83] Cutini P.H., Campelo A.E., Massheimer V.L. (2014). Differential regulation of endothelium behavior by progesterone and medroxyprogesterone acetate. J. Endocrinol..

[84] Simoncini T., Caruso A., Garibaldi S., Fu X.D., Giretti M.S., Baldacci C., Scorticati C., Fornari L., Mannella P., Genazzani A.R. (2006). Activation of nitric oxide synthesis in human endothelial cells using nomegestrol acetate. Obstet. Gynecol..

[85] Oishi A., Takahashi K., Ohmichi M., Mochizuki Y., Inaba N., Kurachi H. (2011). Role of glucocorticoid receptor in the inhibitory effect of medroxyprogesterone acetate on the estrogen-induced endothelial nitric oxide synthase phosphorylation in human umbilical vein endothelial cells. Fertil. Steril..

[86] Houshdaran S., Chen J.C., Vallvé-Juanico J., Balayan S., Vo K.C., Smith-McCune K., Greenblatt R.M., Irwin J.C., Giudice L.C. (2020). Progestins Related to Progesterone and Testosterone Elicit Divergent Human Endometrial Transcriptomes and Biofunctions. Int. J. Mol. Sci..

[87] Truong T.H., Lange C.A. (2018). Deciphering Steroid Receptor Crosstalk in Hormone-Driven Cancers. Endocrinology.

[88] Pang Y., Dong J., Thomas P. (2015). Progesterone increases nitric oxide synthesis in human vascular endothelial cells through activation of membrane progesterone receptor-α. Am. J. Physiol. Endocrinol. Metab..

[89] Dong J., Grunstein J., Tejada M., Peale F., Frantz G., Liang W.C., Bai W., Yu L., Kowalski J., Liang X. (2004). VEGF-null cells require PDGFR alpha signaling-mediated stromal fibroblast recruitment for tumorigenesis. EMBO J..

[90] Soares R., Guerreiro S., Botelho M. (2007). Elucidating progesterone effects in breast cancer: Cross talk with PDGF signaling pathway in smooth muscle cell. J. Cell. Biochem..

[91] Iruela-Arispe M.L., Porter P., Bornstein P., Sage E.H. (1996). Thrombospondin-1, an inhibitor of angiogenesis, is regulated by progesterone in the human endometrium. J. Clin. Investig..

[92] Hyder S.M., Liang Y., Wu J., Welbern V. (2009). Regulation of thrombospondin-1 by natural and synthetic progestins in human breast cancer cells. Endocr. Relat. Cancer.

[93] Arroyo J.A., Winn V.D. (2008). Vasculogenesis and angiogenesis in the IUGR placenta. Semin. Perinatol..

[94] Hyder S.M., Stancel G.M. (1999). Regulation of angiogenic growth factors in the female reproductive tract by estrogens and progestins. Mol. Endocrinol..

[95] Esmailzadeh S., Faramarzi M. (2007). Endometrial thickness and pregnancy outcome after intrauterine insemination. Fertil. Steril..

[96] Jabbour H.N., Kelly R.W., Fraser H.M., Critchley H.O. (2006). Endocrine regulation of menstruation. Endocr. Rev..

[97] Di Renzo G.C., Giardina I., Clerici G., Brillo E., Gerli S. (2016). Progesterone in normal and pathological pregnancy. Horm. Mol. Biol. Clin. Investig..

[98] Shifren J.L., Tseng J.F., Zaloudek C.J., Ryan I.P., Meng Y.G., Ferrara N., Jaffe R.B., Taylor R.N. (1996). Ovarian steroid regulation of vascular endothelial growth factor in the human endometrium: Implications for angiogenesis during the menstrual cycle and in the pathogenesis of endometriosis. J. Clin. Endocrinol. Metab..

[99] Greb R.R., Heikinheimo O., Williams R.F., Hodgen G.D., Goodman A.L. (1997). Vascular endothelial growth factor in primate endometrium is regulated by oestrogen-receptor and progesterone-receptor ligands in vivo. Hum. Reprod..

[100] Matsubara K., Abe E., Matsubara Y., Kameda K., Ito M. (2006). Circulating endothelial progenitor cells during normal pregnancy and pre-eclampsia. Am. J. Reprod. Immunol..

[101] Asahara T., Masuda H., Takahashi T., Kalka C., Pastore C., Silver M., Kearne M., Magner M., Isner J.M. (1999). Bone marrow origin of endothelial progenitor cells responsible for postnatal vasculogenesis in physiological and pathological neovascularization. Circ. Res..

[102] Matsubara Y., Matsubara K. (2012). Estrogen and progesterone play pivotal roles in endothelial progenitor cell proliferation. Reprod. Biol. Endocrinol. RB E.

[103] Gambino L.S., Wreford N.G., Bertram J.F., Dockery P., Lederman F., Rogers P.A. (2002). Angiogenesis occurs by vessel elongation in proliferative phase human endometrium. Hum. Reprod..

[104] Abberton K.M., Healy D.L., Rogers P.A. (1999). Smooth muscle alpha actin and myosin heavy chain expression in the vascular smooth muscle cells surrounding human endometrial arterioles. Hum. Reprod..

[105] Abberton K.M., Taylor N.H., Healy D.L., Rogers P.A. (1999). Vascular smooth muscle cell proliferation in arterioles of the human endometrium. Hum. Reprod..

[106] Kohnen G., Campbell S., Jeffers M.D., Cameron I.T. (2000). Spatially regulated differentiation of endometrial vascular smooth muscle cells. Hum. Reprod..

[107] Girling J.E., Lederman F.L., Walter L.M., Rogers P.A. (2007). Progesterone, but not estrogen, stimulates vessel maturation in the mouse endometrium. Endocrinology.

[108] Bailey D.W., Dunlap K.A., Frank J.W., Erikson D.W., White B.G., Bazer F.W., Burghardt R.C., Johnson G.A. (2010). Effects of long-term progesterone on developmental and functional aspects of porcine uterine epithelia and vasculature: Progesterone alone does not support development of uterine glands comparable to that of pregnancy. Reproduction.

[109] Wen L., Chen L.H., Li H.Y., Chang S.P., Liao C.Y., Tsui K.H., Sung Y.J., Chao K.C. (2009). Roles of estrogen and progesterone in endometrial hemodynamics and vascular endothelial growth factor production. J. Chin. Med. Assoc..

[110] Tan W., Chen L., Guo L., Ou X., Xie D., Quan S. (2014). Relationship between macrophages in mouse uteri and angiogenesis in endometrium during the peri-implantation period. Theriogenology.

[111] Ramathal C.Y., Bagchi I.C., Taylor R.N., Bagchi M.K. (2010). Endometrial decidualization: Of mice and men. Semin. Reprod. Med..

[112] Chen X., Jin X., Liu L., Man C.W., Huang J., Wang C.C., Zhang S., Li T.C. (2015). Differential expression of vascular endothelial growth factor angiogenic factors in different endometrial compartments in women who have an elevated progesterone level before oocyte retrieval, during in vitro fertilization-embryo transfer treatment. Fertil. Steril..

[113] Douglas N.C., Tang H., Gomez R., Pytowski B., Hicklin D.J., Sauer C.M., Kitajewski J., Sauer M.V., Zimmermann R.C. (2009). Vascular endothelial growth factor receptor 2 (VEGFR-2) functions to promote uterine decidual angiogenesis during early pregnancy in the mouse. Endocrinology.

[114] Kapiteijn K., Koolwijk P., van der Weiden R.M., van Nieuw Amerongen G., Plaisier M., van Hinsbergh V.W., Helmerhorst F.M. (2006). Human embryo-conditioned medium stimulates in vitro endometrial angiogenesis. Fertil. Steril..

[115] Manolea M.M., Dijmărescu A.L., Popescu F.C., Novac M.B., DiŢescu D. (2015). Evaluation of the implantation site morphology in spontaneous abortion. Rom. J. Morphol. Embryol..

[116] Lydon J.P., DeMayo F.J., Funk C.R., Mani S.K., Hughes A.R., Montgomery C.A., Shyamala G., Conneely O.M., O’Malley B.W. (1995). Mice lacking progesterone receptor exhibit pleiotropic reproductive abnormalities. Genes Dev..

[117] Johannisson E., Oberholzer M., Swahn M.L., Bygdeman M. (1989). Vascular changes in the human endometrium following the administration of the progesterone antagonist RU 486. Contraception.

[118] Slayden O.D., Zelinski-Wooten M.B., Chwalisz K., Stouffer R.L., Brenner R.M. (1998). Chronic treatment of cycling rhesus monkeys with low doses of the antiprogestin ZK 137 316: Morphometric assessment of the uterus and oviduct. Hum. Reprod..

[119] Fang Z., Yang S., Lydon J.P., DeMayo F., Tamura M., Gurates B., Bulun S.E. (2004). Intact progesterone receptors are essential to counteract the proliferative effect of estradiol in a genetically engineered mouse model of endometriosis. Fertil. Steril..

[120] Duran C.L., Abbey C.A., Bayless K.J. (2018). Establishment of a three-dimensional model to study human uterine angiogenesis. Mol. Hum. Reprod..

[121] Rashidi B., Mardani M., Karizbodagh M.P. (2017). Evaluation of Progesterone and Ovulation-stimulating Drugs on the Glandular Epithelium and Angiogenesis in Mice. Adv. Biomed. Res..

[122] Subakir S.B., Hadisaputra W., Handoyo A.E., Affandi B. (1996). Endometrial angiogenic response in Norplant users. Hum. Reprod..

[123] Goodger A.M., Rogers P.A., Affandi B. (1994). Endometrial endothelial cell proliferation in long-term users of subdermal levonorgestrel. Hum. Reprod..

[124] Vázquez F., Rodríguez-Manzaneque J.C., Lydon J.P., Edwards D.P., O’Malley B.W., Iruela-Arispe M.L. (1999). Progesterone regulates proliferation of endothelial cells. J. Biol. Chem..

[125] Hsu S.P., Ho P.Y., Juan S.H., Liang Y.C., Lee W.S. (2008). Progesterone inhibits human endothelial cell proliferation through a p53-dependent pathway. Cell. Mol. Life Sci..

[126] Hsu S.P., Lee W.S. (2011). Progesterone receptor activation of extranuclear signaling pathways in regulating p53 expression in vascular endothelial cells. Mol. Endocrinol..

[127] Kim I.D., Ahn K.H., Lee S., Hong S.C., Kim S.H., Kim T. (2013). Effect of ovariectomy, 17-beta estradiol, and progesterone on histology and estrogen receptors of bladder in female partial bladder outlet obstruction rat model. J. Obstet. Gynaecol. Res..

[128] Katayama Y., Uchino J., Chihara Y., Tamiya N., Kaneko Y., Yamada T., Takayama K. (2019). Tumor Neovascularization and Developments in Therapeutics. Cancers.

[129] Troisi J., Sarno L., Landolfi A., Scala G., Martinelli P., Venturella R., Di Cello A., Zullo F., Guida M. (2018). Metabolomic Signature of Endometrial Cancer. J. Proteome Res..

[130] Kim J.J., Kurita T., Bulun S.E. (2013). Progesterone action in endometrial cancer, endometriosis, uterine fibroids, and breast cancer. Endocr. Rev..

[131] Ramirez P.T., Frumovitz M., Bodurka D.C., Sun C.C., Levenback C. (2004). Hormonal therapy for the management of grade 1 endometrial adenocarcinoma: A literature review. Gynecol. Oncol..

[132] Lee I.I., Maniar K., Lydon J.P., Kim J.J. (2016). Akt regulates progesterone receptor B-dependent transcription and angiogenesis in endometrial cancer cells. Oncogene.

[133] Hyder S.M., Murthy L., Stancel G.M. (1998). Progestin regulation of vascular endothelial growth factor in human breast cancer cells. Cancer Res..

[134] Fujimoto J., Sakaguchi H., Hirose R., Ichigo S., Tamaya T. (1999). Progestins suppress estrogen-induced expression of vascular endothelial growth factor (VEGF) subtypes in uterine endometrial cancer cells. Cancer Lett..

[135] Mohr P.E., Wang D.Y., Gregory W.M., Richards M.A., Fentiman I.S. (1996). Serum progesterone and prognosis in operable breast cancer. Br. J. Cancer.

[136] Fu X.D., Russo E., Zullino S., Genazzani A.R., Simoncini T. (2010). Sex steroids and breast cancer metastasis. Horm. Mol. Biol. Clin. Investig..

[137] Wu J., Brandt S., Hyder S.M. (2005). Ligand- and cell-specific effects of signal transduction pathway inhibitors on progestin-induced vascular endothelial growth factor levels in human breast cancer cells. Mol. Endocrinol..

[138] Thomassen M., Tan Q., Kruse T.A. (2008). Gene expression meta-analysis identifies metastatic pathways and transcription factors in breast cancer. BMC Cancer.

[139] Kougioumtzi A., Tsaparas P., Magklara A. (2014). Deep sequencing reveals new aspects of progesterone receptor signaling in breast cancer cells. PLoS ONE.

[140] Lee O., Choi M.R., Christov K., Ivancic D., Khan S.A. (2016). Progesterone receptor antagonism inhibits progestogen-related carcinogenesis and suppresses tumor cell proliferation. Cancer Lett..

[141] Carroll C.E., Liang Y., Benakanakere I., Besch-Williford C., Hyder S.M. (2013). The anticancer agent YC-1 suppresses progestin-stimulated VEGF in breast cancer cells and arrests breast tumor development. Int. J. Oncol..

[142] Wu J., Richer J., Horwitz K.B., Hyder S.M. (2004). Progestin-dependent induction of vascular endothelial growth factor in human breast cancer cells: Preferential regulation by progesterone receptor B. Cancer Res..

[143] Murphy B.L., Day C.N., Hoskin T.L., Habermann E.B., Boughey J.C. (2019). Adolescents and Young Adults with Breast Cancer have More Aggressive Disease and Treatment Than Patients in Their Forties. Ann. Surg. Oncol..

[144] Vameşu S. (2007). Angiogenesis and progesterone receptor status in primary breast cancer patients: An analysis of 158 needle core biopsies. Rom. J. Morphol. Embryol..

[145] Uchida M., Tsuboi H., Yamaji T., Murata N., Kohno T., Sugino E., Hibino S., Shimamura M., Oikawa T. (2000). Inhibition by 9alpha-fluoromedoroxyprogesterone acetate (FMPA) against mammary carcinoma induced by dimethylbenz[a]anthracene in rats and angiogenesis in the rabbit cornea-comparison with medroxyprogesterone acetate (MPA). Cancer Lett..

[146] Pietras R.J., Márquez D.C., Chen H.W., Tsai E., Weinberg O., Fishbein M. (2005). Estrogen and growth factor receptor interactions in human breast and non-small cell lung cancer cells. Steroids.

[147] Tan J., Paria B.C., Dey S.K., Das S.K. (1999). Differential uterine expression of estrogen and progesterone receptors correlates with uterine preparation for implantation and decidualization in the mouse. Endocrinology.

[148] Mirkin S., Archer D.F. (2004). Effects of levonorgestrel, medroxyprogesterone acetate, norethindrone, progesterone, and 17beta-estradiol on thrombospondin-1 mRNA in Ishikawa cells. Fertil. Steril..

[149] Classen-Linke I., Alfer J., Krusche C.A., Chwalisz K., Rath W., Beier H.M. (2000). Progestins, progesterone receptor modulators, and progesterone antagonists change VEGF release of endometrial cells in culture. Steroids.

[150] Mangal R.K., Wiehle R.D., Poindexter A.N., Weigel N.L. (1997). Differential expression of uterine progesterone receptor forms A and B during the menstrual cycle. J. Steroid Biochem. Mol. Biol..

[151] Wang H., Critchley H.O., Kelly R.W., Shen D., Baird D.T. (1998). Progesterone receptor subtype B is differentially regulated in human endometrial stroma. Mol. Hum. Reprod..

[152] Jacobsen B.M., Richer J.K., Schittone S.A., Horwitz K.B. (2002). New human breast cancer cells to study progesterone receptor isoform ratio effects and ligand-independent gene regulation. J. Biol. Chem..

[153] Heryanto B., Rogers P.A. (2002). Regulation of endometrial endothelial cell proliferation by oestrogen and progesterone in the ovariectomized mouse. Reproduction.

[154] Li Y., Adur M.K., Kannan A., Davila J., Zhao Y., Nowak R.A., Bagchi M.K., Bagchi I.C., Li Q. (2016). Progesterone Alleviates Endometriosis via Inhibition of Uterine Cell Proliferation, Inflammation and Angiogenesis in an Immunocompetent Mouse Model. PLoS ONE.

[155] Shao R., Cao S., Wang X., Feng Y., Billig H. (2014). The elusive and controversial roles of estrogen and progesterone receptors in human endometriosis. Am. J. Transl. Res..

[156] Benakanakere I., Besch-Williford C., Schnell J., Brandt S., Ellersieck M.R., Molinolo A., Hyder S.M. (2006). Natural and synthetic progestins accelerate 7,12-dimethylbenz[a]anthracene-initiated mammary tumors and increase angiogenesis in Sprague-Dawley rats. Clin. Cancer Res..

[157] Finlay-Schultz J., Gillen A.E., Brechbuhl H.M., Ivie J.J., Matthews S.B., Jacobsen B.M., Bentley D.L., Kabos P., Sartorius C.A. (2017). Breast Cancer Suppression by Progesterone Receptors Is Mediated by Their Modulation of Estrogen Receptors and RNA Polymerase III. Cancer Res..

[158] Valadez-Cosmes P., Vázquez-Martínez E.R., Cerbón M., Camacho-Arroyo I. (2016). Membrane progesterone receptors in reproduction and cancer. Mol. Cell Endocrinol..

[159] Ruan X., Cai G., Wei Y., Gu M., Zhang Y., Zhao Y., Mueck A.O. (2020). Association of circulating Progesterone Receptor Membrane Component-1 (PGRMC1) with breast tumor characteristics and comparison with known tumor markers. Menopause.

[160] Zhang Y., Ruan X., Willibald M., Seeger H., Fehm T., Neubauer H., Mueck A.O. (2016). May progesterone receptor membrane component 1 (PGRMC1) predict the risk of breast cancer?. Gynecol. Endocrinol. Off. J. Int. Soc. Gynecol. Endocrinol..

[161] Asperger H., Stamm N., Gierke B., Pawlak M., Hofmann U., Zanger U.M., Marton A., Katona R.L., Buhala A., Vizler C. (2020). Progesterone receptor membrane component 1 regulates lipid homeostasis and drives oncogenic signaling resulting in breast cancer progression. Breast Cancer Res..

[162] Pedroza D.A., Subramani R., Tiula K., Do A., Rashiraj N., Galvez A., Chatterjee A., Bencomo A., Rivera S., Lakshmanaswamy R. (2021). Crosstalk between progesterone receptor membrane component 1 and estrogen receptor α promotes breast cancer cell proliferation. Lab. Investig..

[163] Hewitt S.C., Korach K.S. (2000). Progesterone action and responses in the alphaERKO mouse. Steroids.

[164] Giulianelli S., Vaqué J.P., Soldati R., Wargon V., Vanzulli S.I., Martins R., Zeitlin E., Molinolo A.A., Helguero L.A., Lamb C.A. (2012). Estrogen receptor alpha mediates progestin-induced mammary tumor growth by interacting with progesterone receptors at the cyclin D1/MYC promoters. Cancer Res..

[165] Schneck H., Ruan X., Seeger H., Cahill M.A., Fehm T., Mueck A.O., Neubauer H. (2013). Membrane-receptor initiated proliferative effects of dienogest in human breast cancer cells. Gynecol. Endocrinol. Off. J. Int. Soc. Gynecol. Endocrinol..

[166] Susa T., Ikaga R., Kajitani T., Iizuka M., Okinaga H., Tamamori-Adachi M., Okazaki T. (2015). Wild-type and specific mutant androgen receptor mediates transcription via 17β-estradiol in sex hormone-sensitive cancer cells. J. Cell Physiol..

[167] Sato B. (1999). Can an autocrine loop explain sex-hormone-dependent tumor growth? A brief overview. Oncology.

[168] Guzeloglu Kayisli O., Kayisli U.A., Basar M., Semerci N., Schatz F., Lockwood C.J. (2015). Progestins Upregulate FKBP51 Expression in Human Endometrial Stromal Cells to Induce Functional Progesterone and Glucocorticoid Withdrawal: Implications for Contraceptive- Associated Abnormal Uterine Bleeding. PLoS ONE.

[169] Piasecka D., Braun M., Kitowska K., Mieczkowski K., Kordek R., Sadej R., Romanska H. (2019). FGFs/FGFRs-dependent signalling in regulation of steroid hormone receptors-implications for therapy of luminal breast cancer. J. Exp. Clin. Cancer Res..

[170] Pan C., Liu Y.P., Li Y.F., Hu J.X., Zhang J.P., Wang H.M., Li J., Xu L.C. (2012). Effects of cypermethrin on the ligand-independent interaction between androgen receptor and steroid receptor coactivator-1. Toxicology.

[171] Robertson S., Rohwer J.M., Hapgood J.P., Louw A. (2013). Impact of glucocorticoid receptor density on ligand-independent dimerization, cooperative ligand-binding and basal priming of transactivation: A cell culture model. PLoS ONE.

[172] Zhang G., Yanamala N., Lathrop K.L., Zhang L., Klein-Seetharaman J., Srinivas H. (2010). Ligand-independent antiapoptotic function of estrogen receptor-beta in lung cancer cells. Mol. Endocrinol..

[173] Mitani Y., Lin S.H., Pytynia K.B., Ferrarotto R., El-Naggar A.K. (2020). Reciprocal and Autonomous Glucocorticoid and Androgen Receptor Activation in Salivary Duct Carcinoma. Clin. Cancer Res..

[174] Hardy D.B., Janowski B.A., Chen C.C., Mendelson C.R. (2008). Progesterone receptor inhibits aromatase and inflammatory response pathways in breast cancer cells via ligand-dependent and ligand-independent mechanisms. Mol. Endocrinol..

[175] Xiao J., Chen X., Lu X., Xie M., He B., He S., You S., Chen Q. (2020). Progesterone/Org inhibits lung adenocarcinoma cell growth via membrane progesterone receptor alpha. Thorac. Cancer.

